# Transplantation of adipose-derived stem cells ameliorates *Echinococcus multilocularis*-induced liver fibrosis in mice

**DOI:** 10.1371/journal.pntd.0010175

**Published:** 2022-01-31

**Authors:** Ning Yang, Wenmei Ma, Ying Ke, Hui Liu, Jin Chu, Li Sun, Guodong Lü, Xiaojuan Bi, Renyong Lin

**Affiliations:** 1 State Key Laboratory of Pathogenesis, Prevention and Treatment of High Incidence Diseases in Central Asia, Clinical Medical Research Institute, the First Affiliated Hospital of Xinjiang Medical University, Urumqi, Xinjiang, China; 2 Pathology department, the First Affiliated Hospital of Xinjiang Medical University, Urumqi, Xinjiang, China; 3 Graduate School, Xinjiang Medical University, Urumqi, Xinjiang, China; 4 Xinjiang Key Laboratory of Echinococcosis, the First Affiliated Hospital of Xinjiang Medical University, Urumqi, Xinjiang, China; 5 Basic Medical College, Xinjiang Medical University, Urumqi, Xinjiang, China; Instituto de Salud Carlos III, SPAIN

## Abstract

**Background:**

Alveolar echinococcosis (AE) can cause severe liver fibrosis and could be fatal if left untreated. Currently, there are no effective therapeutic options for AE-induced liver fibrosis. In view of the therapeutic potential of adipose-derived stem cells (ADSCs), we investigated whether ADSCs transplantation has the ability to control or reverse fibrosis progression in the liver of *Echinococcus multilocularis* (*E*. *multilocularis*) infected mice.

**Methodology/Principal findings:**

C57BL/6 mice infected with *E*. *multilocularis* through portal vein inoculation were intravenously injected with ADSCs isolated from inguinal adipose tissues of 6–8 weeks old mice. Histopathological analysis including heamatoxylin & eosin staining as well as Masson’s trichrome staining, and Sirius red staining were performed to access the degree of liver fibrosis. Histopathological examination 30 days after ADSCs transplantation revealed that ADSCs significantly decreased the degree of liver fibrosis in *E*. *multilocularis* infected mice by inhibiting the expressions of α-SMA and type 1 collagen deposition. In addition, compared to the non-transplanted group, ADSCs transplantation reduced fibrotic areas in *E*. *multilocularis* infected mice. We also found that ADSCs transplantation significantly down-regulated TGF-β1 and TGF-βR expressions, while up-regulating Smad7 expression in the TGF-β/Smad signaling pathway.

**Conclusions:**

ADSCs can alleviate *Echinococcus multilocularis* infection-induced liver fibrosis by modulating the activity level of the TGF-β/Smad7 signaling pathway and provide a potential therapeutic approach for *E*. *multilocularis*-induced fibrosis.

## Author summary

Alveolar echinococcosis (AE), one of the most deadly zoonoses in both developing and developed Northern Hemisphere countries, is caused by the larval stages (metacestode) of the tapeworm *Echinococcus multilocularis* (*E*. *multilocularis*). This disease is characterized by the development of severe fibrosis around the lesion, which gradually replaces normal liver parenchyma and affects liver function. Mesenchymal stem cells (MSCs) have been evaluated as an alternative therapeutic option for tissue repair (including fibrosis). Adipose-derived stem cells (ADSCs), which are one of MSCs subsets, can easily obtained from adipose tissues through liposuction, implying that a large number of cells can be obtained with less traumatic surgery. In multiple tissues, including pulmonary, dermal, renal, and especially liver tissues, ADSCs have been shown to be effective in fibrosis treatment. Therefore, we investigated the therapeutic potential of ADSCs on *Echinococcus multilocularis* infections. Briefly, *E*. *multilocularis*-infected mice that were transplanted with ADSCs were found to have less liver fibrosis, indicating the potential of ADSCs as novel therapeutic approaches for *E*. *multilocularis*-induced liver fibrosis. Although these findings are promising, studies using long-term AE infection models should be performed to confirm the therapeutic effects of ADSCs on AE.

## Introduction

Alveolar echinococcosis (AE), one of the most dangerous zoonoses in both developing and developed Northern Hemisphere countries, is caused by infection with larval stages (metacestode) of the tapeworm *Echinococcus multilocularis* (*E*. *multilocularis*) [1,2]. If left untreated, AE has a very high mortality rage (> 90%) [3]. The continuous growth of metacestodes in liver tissues of their intermediate hosts obstructs blood vessels and bile ducts, leading to organ failure [4]. The development of severe fibrosis surrounding the lesion is a feature of AE, and excess fibrosis can damage the liver parenchyma. Eventually, the collapsed parenchyma is replaced by collagen-rich tissues, resulting in the loss of liver function [5]. We previously found that the fibrotic "barrier" at the periphery of the lesion constantly erodes the surrounding liver parenchyma, leading to death of the host [6]. Presently, although hepatectomy is the sole curative treatment option for AE, it is not suitable for patients with severe disease in whom the lesions surpass the resectability threshold or those with decompensated liver dysfunction caused by AE. In terms of pharmaceutical treatment, the fibrotic "barrier" around the lesion limits the efficacy of benzimidazole-based chemotherapeutic drugs [7]. Therefore, there is an urgent need to develop new therapeutic approaches for *E*. *multilocularis*-induced liver fibrosis, thereby minimizing liver damage and allowing antiparasitic drugs to effectively penetrate the lesion.

Mesenchymal stem cells (MSCs), which are stromal cell progenitor cells with immunosuppressive properties and abilities to differentiate mesoderm cell lines are currently attracting significant attention from researchers due to their broad-ranging clinical therapeutic potential (including fibrosis) [8,9]. Adipose-derived stem cells (ADSCs), which are one of the MSCs subsets can be easily obtained from adipose tissues by liposuction, implying that a large number of cells can be obtained with less traumatic surgerical processes [10]. ADSCs are beneficial for the treatment of fibrosis in various tissues, including pulmonary [11], dermal [12], renal [13], and the liver [14–17]. However, the significance of ADSCs for AE-induced fibrosis has not been documented. Therefore, we investigated the therapeutic potential of ADSCs on *E*. *multilocularis*-induced liver fibrosis.

In recent years, anti-fibrotic mechanisms of ADSCs have been progressively elucidated [18,19]. TGF-β is one of the most classical and important signaling pathway in fibrosis pathogenesis, and its up-regulation activates hepatic stellate cells (HSCs), α-smooth muscle actin (α-SMA) expression, and the synthesis of ECM proteins, such as collagens [20]. Moreover, the TGF-β signaling pathway mediates the anti-fibrotic effects of ADSCs in diseases. In acute radiation-induced lung injury, ADSCs transplantation have been shown to inhibit lung fibrosis by suppressing TGF-β1 expression. In addition, ADSCs transplantation suppressed the expression of fibrotic markers (CTGF, α-SMA, and Col1A1) as well as hydroxyproline levels [21]. Li et al. [22] reported that ADSCs transplantation inhibited collagen deposition and α–SMA expression by regulating of the p38/MAPK signaling pathway. Sun et al. [23] documented that ADSCs transplantation alleviated irradiation-induced fibrosis by suppressing TGF-β1 levels in irradiated skeletal muscles. However, it has not been documented whether the TGF-β signaling pathway is involved in anti-fibrotic effects of ADSCs in AE. Therefore, we evaluated the potential anti-fibrotic effects and mechanisms of ADSCs in experimental models of AE.

## Methods

### Ethics statement

The animal experiments were approved by the institutional Animal Care and Use Committee and the Ethical Committee of the First Affiliated Hospital of Xinjiang Medical University (No. 20140411–05).

### Isolation and characterization of ADSCs

ADSCs were isolated as previously described [24]. Briefly, inguinal adipose tissues were obtained in a sterile environment from 6–8 weeks old C57BL/6 mice (Beijing Vital River Laboratory Animal Technology Co. Ltd, Beijing, China). The inguinal adipose tissues were shredded and digested with 200 U/ml collagenase type I solution (Worthington, USA) at 37°C for 30 min. After being washed using phosphate buffer saline (PBS, 1x, pH7.4) containing 1000 U/ml penicillin and streptomycin, cells were grown in Dulbecco’s modified Eagle’s medium (DMEM, low glucose, Hyclone, USA) containing 10% fetal bovine serum (FBS, Gibco, USA), 2 mM L-glutamine (Hyclone, USA), 100 U/ml penicillin, and streptomycin penicillin (Hyclone, USA). After 24 h of adhesion, the media was refreshed every 2–3 days until cells reached 85% confluence. Subsequently, cells were washed twice using PBS, followed by the addition of 1-2ml 0.25% trypsin, and digested at 37°C for 2 min. FBS were added to terminate the reaction after which the solution was centrifuged at 1200 rpm for 5 min. Then, cells were cultured at a density of 4×10^4^/ml.

The ability of ADSCs to differentiate into osteogenic and adipogenic cells has been reported [25]. Before induction, third-passaged ADSCs were seeded at a density of 4×10^3^ cells/cm^2^ until 70% confluent. For osteogenic induction, ADSCs were cultured in an osteogenic induction media (L-DMEM medium supplemented with 10% fetal bovine serum, 0.1 μmol/L dexamethasones, 50 μmol/L ascorbic acid, and 10 mmol/L sodium β-glycerophosphate). When calcium deposition was observed in the dish, induction was terminated. Then, cells were washed twice using PBS (1x, pH 7.4) and fixed in 4% (v/v) paraformaldehyde for 30 min at room temperature. Cells were stained with Alizarin Red S after which the density of calcium nodules was determined using a microscope (Leica DM6000B, Germany). For adipogenic induction, ADSCs were cultured in an adipogenic induction media (L-DMEM medium supplemented with 10% fetal bovine serum, 1 μmol/L dexamethasone, 200 μmol/L indomethacin, 500 μmol/L IBMX, and 10 μmol/L insulin). When lipid droplets formed in more than 70% of cells in the plate, induction was stopped. Then, the cells were washed twice using PBS (1x, pH 7.4) and fixed in 4% (v/v) paraformaldehyde for 30 min at room temperature. Oil Red O staining was performed to identify intracellular lipid droplets, which were imaged under a microscope (Leica DM6000B, Germany). These experiments were perfomed in triplicates.

### Flow cytometry analysis

Flow cytometry (FACS AriaII, BD, USA) was used to confirm the identity of ADSCs by detecting the expressions of cell surface markers, including CD29-FITC (102205, Biolegend), CD90-APC-Cy7 (561641, BD), CD105-BB515 (564744, BD), CD44-PE-Cy5 (103009, Biolegend), CD31-APC (551262, BD), CD34-FITC(11-0341-82, eBioscience), and CD45-APC (559864, BD) [25]. Third-passage ADSCs were digested with trypsin (Sigma-Aldrich, United States), re-suspended in PBS, and counted using a hemocytometer. A total of 1×10^6^ cells were treated with the fluorochrome-conjugated antibody at a dilution ratio of 1:10 for 30 min at room temperature with protection from light. **S1 Table** shows the antibodies used in these experiments.

### Animal experiments

Eighteen healthy female C57BL/6 mice (weighing about 18-20g, Beijing Vital River Laboratory Animal Technology Co. Ltd, Beijing, China) were randomized into three groups (sham operation + vehicle injection (Sham group), *E*. *multilocularis* infection + vehicle injection (Em group), and *E*. *multilocularis* infection + ADSCs transplantation (Em + ADSCs group)) of six mice each. The temperature in the pathogen-free feeding was maintained at 20–24°C under a 12 h light/dark cycle. Before the experiment, mice had free access to a regular diet and water for one week.

### Establishment of *E*. *multilocularis* infection models

Mice in *E*. *multilocularis* infection groups were inoculated with protoscoleces suspended in normal saline via the hepatic portal vein, as previously described [6], while mice in the sham operation group were injected with equivalent amount of normal saline. Briefly, protoscoleces were obtained from intraperitoneal lesions maintained in Mongolian gerbils, washed several times using PBS, counted under a microscope, and adjusted to the appropriate concentrations before injection. Then, mice were anesthetized using isoflurane and injected with protoscoleces (3000 PSCs/mouse in 200μl normal saline) or 200 μl normal saline via the hepatic portal vein, respectively.

### CM-DiI labeling and ADSCs transplantation

**Fig 1:**
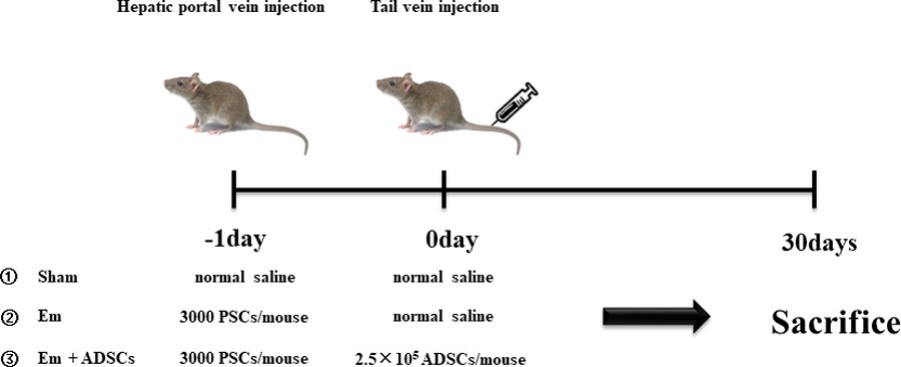
Schematic presentation of animal experimental protocol. PSCs, protoscoleces; ADSCs, adipose-derived stem cells.

Third-passaged ADSCs were labeled with DiI (42364, Sigma–Aldrich) to trace transplanted cells in the liver as previously described [26]. ADSCs were digested, resuspended in a media supplemented with DiI (final concentration at 5 μg/ml) and incubated at 37°C for 20 min under light protection. After incubation, cells were centrifuged at 6000 rpm for 2 min. Fluorescence microscopy was used to assess labeling efficiencies, while cell viabilities were determined by staining with 0.2% trypan blue. These assays were repeated in triplicates. The day after surgery, mice in the Em + ADSCs group were transplanted with ADSCs (2.5×10^5^ ADSCs/ mouse) via tail vein injection as shown in **Fig 1**. Mice in the Sham or Em groups were given equivalent amount of normal saline.

### Histopathological and Immunohistochemical analysis

At 30 days post-transplantation, liver samples were harvested and divided into two parts, one was processed for histopathological assessment while the other was promptly cryopreserved in liquid nitrogen for RNA or protein extraction. For histopathological analyses, samples were fixed in 4% paraformaldehyde for 48 h and embedded in paraffin [17]. Hematoxylin & eosin (H&E) and immunohistochemistry staining were performed on serial sections (4μm thick). In addition, liver fibrosis in mice was assessed using Sirius red and Masson’s trichrome staining as previously described [27]. Light microscopy (DM3000, Leica, Germany) was used to image the Sirius red and Masson’s trichrome stained sections, which were quantitatively analyzed using the Image-Pro Plus software (Version 6.0.0.260, Media Cybernetics, USA). Differences in each group were determined using the positive area of staining.

IHC analysis was performed as previously described [28]. Briefly, paraffin sections were deparaffinized, rehydrated, antigen recovered, and incubated overnight at 4°C in the presence of the following primary antibodies: anti-α-SMA (1:1000, ab124964, Abcam); anti-COL1A1 (1:100,ab34710, Abcam); anti-TGF-β (1:50, SC-130348, Santa Cruz Biotechnology); anti-TGF-β R1 (1:75, ab31013, Abcam) and anti-TGF-β R2 (1:50, ab61213, Abcam). Then, sections were incubated with secondary antibodies (HRP-conjugated goat anti-rabbit IgG, 1:500 (ab97051, Abcam)) at room temperature for 2 h. The HRP/DAB IHC detection system (ab64238, Abcam) was used to detect immunoreactivity. Images for immunohistochemical analyses were acquired by light microscopy (DM3000, Leica, Germany) and quantitatively analyzed using the Image-Pro Plus software (Version 6.0.0.260, Media Cybernetics, USA). α-SMA- and COL1A1-positive areas were measured to determine differences in each group. The average optical density (AOD = IOD/Area) of TGF-β was quantified to determine differences in each group. TGF-β R1 and TGF-β R2-positive cells were counted in three random fields under a high-power field (HPF) to determine differences in each group.

### Quantitative real-time PCR (qRT-PCR) analysis

Total RNA were extracted from liver tissues using the TRIzol reagent (Invitrogen) [17]. Purified total RNA was quantified using Nanodrop ND2000 (NanoDrop Technologies, Wilmington, DE, USA) and reverse-transcribed using a PrimeScript RT reagent Kit (RR047A, Takara, Japan). Then, 2 μl of cDNA was mixed with 18μl of Master Mix (miScript SYBR Green PCR Kit, 218073, Germany, Qiagen) after which RT-PCR was performed using the CFX96 Touch System (BioRad). The 2−ΔCt method was used to calculate relative mRNA expressions which were normalized to the housekeeping gene *GAPDH* [29]. **S2 Table** shows the primers used in this study.

### Western blot analysis

Total proteins were extracted using ice-cold RIPA lysis buffer containing a protease inhibitor cocktail (Invitrogen) [17]. The BCA Protein Assay Kit (23225, Thermo Fisher Scientific, USA) was used to quantify proteins. Then, equal amounts of protein lysate (75 μg) were separated by 10% SDS-PAGE electrophoresis and transferred to a 0.45 μm polyvinylidene fluoride membrane (Millipore). Membranes were blocked using 5% skimmed milk in TBST (pH 7.6) at room temperature for 1 h, and incubated overnight at 4°C in the presence of the following primary antibodies: anti-Smad7 (1:1000, Ab190987, Abcam), and anti-GAPDH (1:10000, ab181602, Abcam). Subsequently, they were incubated with Goat Anti-rabbit IgG H&L/HRP secondary antibody (1:5000, bs-0295G-HRP, Beijing Biosynthesis Biotechnology Co. Ltd, Beijing, China). Enhanced chemiluminescence western blotting reagents (BL520A, Biosharp, Anhui, China) were used for protein detection. Protein density data were normalized to GAPDH levels (n = 6/group) and reported as fold change values ± standard error of the mean.

### Statistical analysis

GraphPad Prism 6.0 (Version 8.0.1, CA, USA) was used for statistical analyses. Residual normality of the data was determined by the Shapiro-Wilk test. Comparisons of means among groups was done by one-way ANOVA followed by Tukey’s post hoc test for multiple comparisons. p ≤0.05 was the threshold for significance.

## Results

### Morphological characteristics and pluripotency of ADSCs

**Fig 2:**
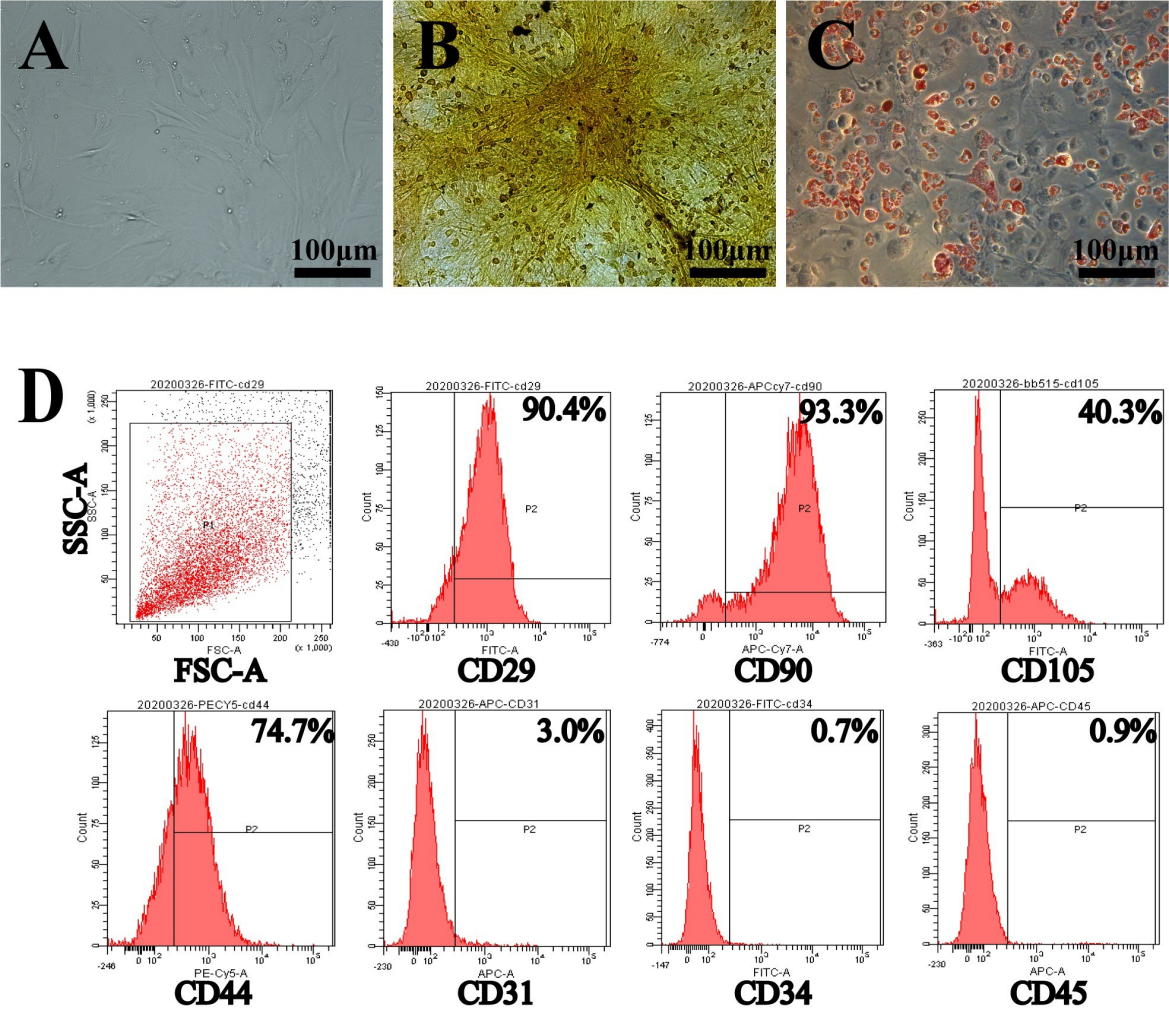
Characterization of ADSCs. A: Morphologies of ADSCs at the 3^rd^ passage. B: Osteogenic differentiation of ADSCs at the 3^rd^ passage. C: Adipogenic differentiation of ADSCs at the 3^rd^ passage. D: Flow cytometric analysis of mouse ADSCs. These experiments were performed in triplicates.

Third-passage ADSCs were adherent and exhibited spindle-shaped morphologies. When ADSCs were maintained in conditioned induction media, they were able to differentiate into adipocytes and osteoblasts. Oil red O staining and alizarin red staining were performed to confirm the differentiation of ADSCs into adipocytes and osteoblasts (**Fig 2A and 2C**). Flow cytometry analysis confirmed the presence of mesenchymal stromal cell surface markers (CD29, CD90, CD44, and CD105) and the absence of epithelial and hematopoietic surface markers (CD31, CD34, and CD45) (**Fig 2D**).

### ADSCs transplantation ameliorated liver fibrosis in *E*. *multilocularis* infected mice

**Fig 3:**
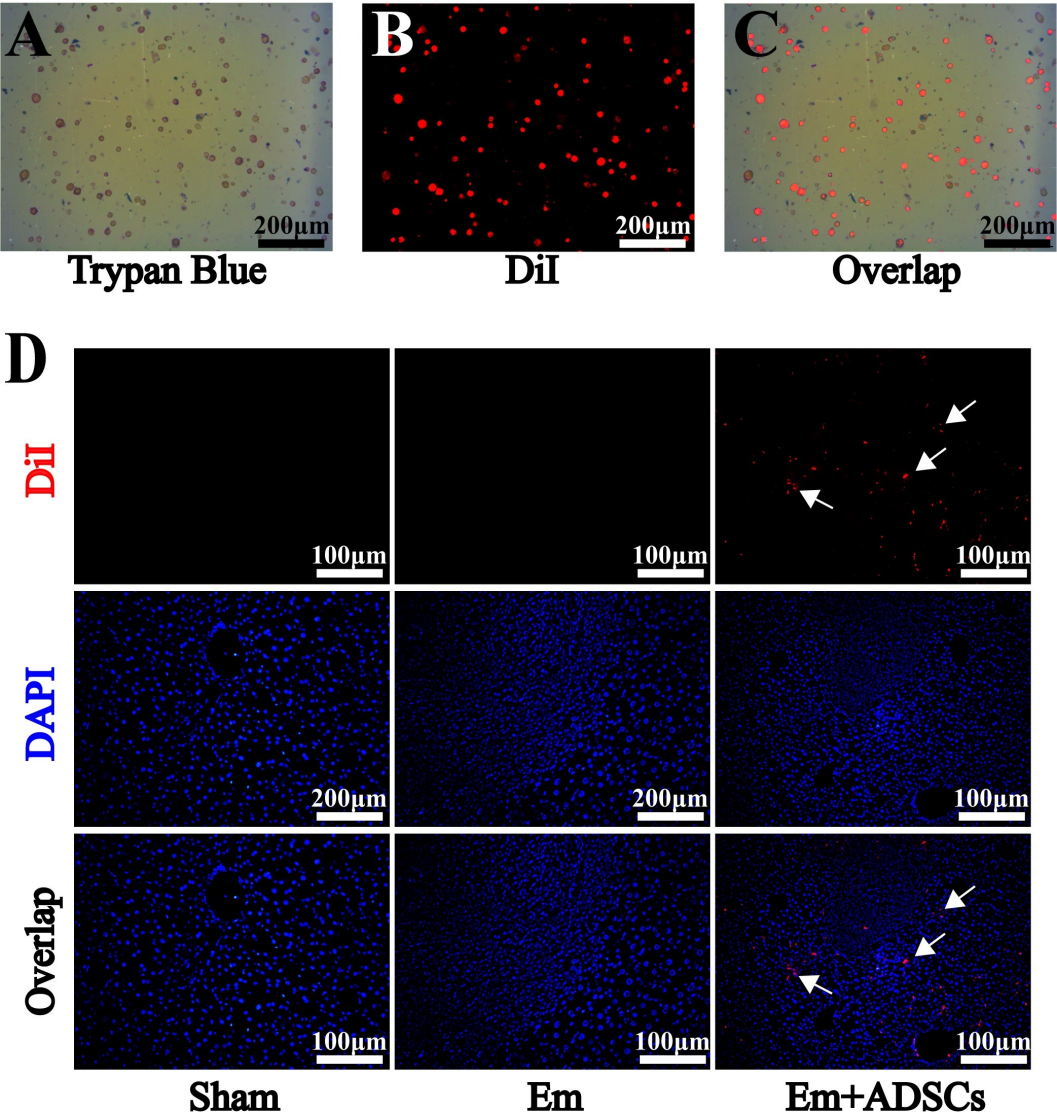
DiI-labeled ADSCs migrated to peripheral regions of the lesion. A: Evaluation of cell viability before ADSCs transplantation. B: Efficiency of ADSCs fluorescently labeled with DiI before transplantation. C: Overlap image of DiI and trypan blue staining. D: DAPI and DiI fluorescence double staining for ADSCs tracing *in vivo*. Data are presented as mean ± SD (n = 6). White arrows indicates CM-DiI-labeled cells.

**Fig 4:**
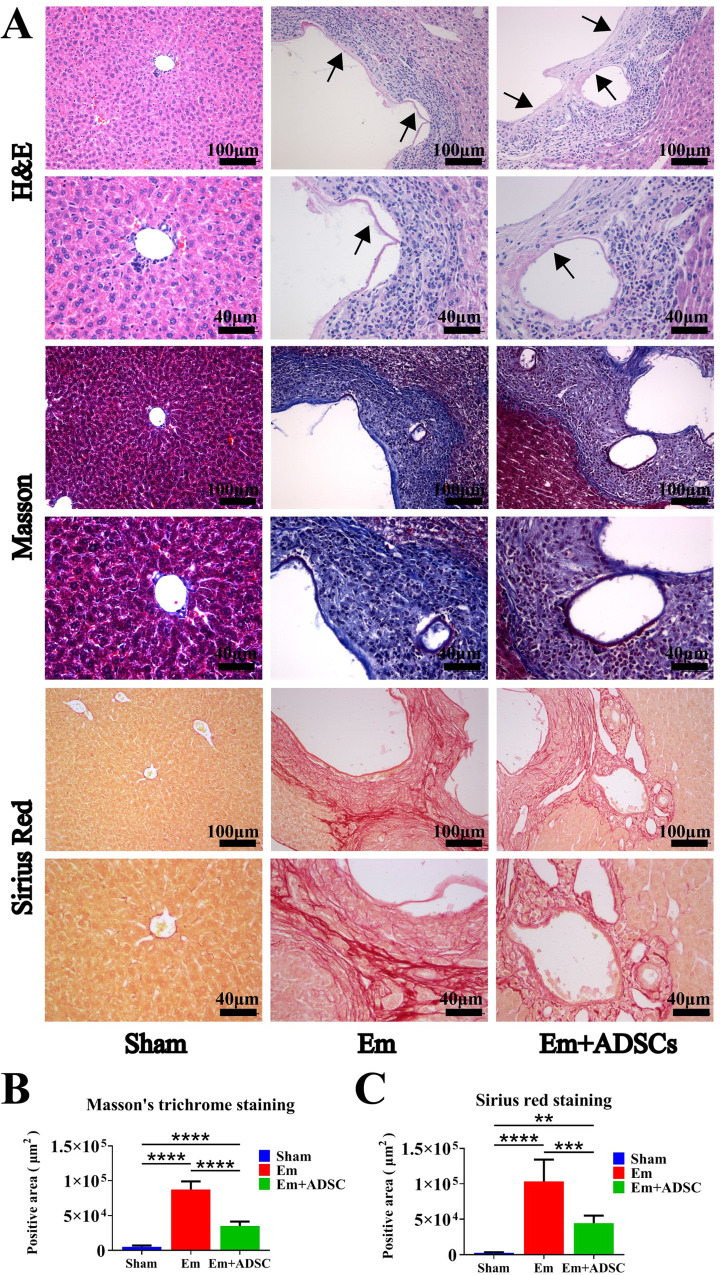
ADSCs transplantation ameliorates liver fibrosis in *E*. *multilocularis* infected mice. A: Representative images of H&E-stained liver sections in each group (top); Representative images of Sirius red stained liver sections in each group (middle); Representative images of Masson’s trichrome stained liver sections in each group (bottom). B: Quantification results of Masson’s trichrome staining. C: Quantification results of Sirius red staining. Data are presented as mean ± SD (n = 6). **p < 0.01, *** *p* < 0.001, **** *p* < 0.0001. Black arrows indicate the germinal layer.

One day after establishing *E*. *multilocularis* infection mice models, CM-DiI labeled ADSCs were transplanted into the mice via tail vein injection. Before transplantation, mean CM-DiI labeling efficiency of ADSCs was 95.3% ± 0.9%, while mean cell viability was 92.3% ± 0.4% (**Fig 3A and 3C**). Thirty days after transplantation, DiI fluorescence was detected in the region surrounding the lesion in the liver of the Em + ADSCs group, but not in other groups (**Fig 3D**). H&E staining showed that morphologies of liver tissues from the Sham group were normal. The Em group had a high frequency of lesions with germinal layers in the liver, as well as severe infiltration of inflammatory cells and fibrosis surrounding the lesion. Furthermore, the Em + ADSCs group had less fibrosis and inflammatory cell infiltration than the Em group (**Fig 4A**). Representative images of metacestode tissues in the livers of mice from different groups showed that ADSCs transplantion reduced the degrees of *E*. *multilocularis* infection (**S1 Fig**). Data were quantified to assess the degree of liver fibrosis, by analyzing fibrotic areas of Sirius red staining and Masson’s trichrome staining. According to quantification results, the fibrotic areas of the livers in the Em group were significantly higher than in the Sham group, however, compared to the Em group, liver fibrosis degrees after ADSCs transplantation were significantly reduced (**Fig 4B**). These findings indicate that ADSCs transplantation effectively alleviated hepatic histological changes and liver fibrosis degrees in mice models of *E*. *multilocularis* infection.

### ADSCs transplantation inhibited HSCs activation and collagen deposition in *E*. *multilocularis* infected mice

**Fig 5:**
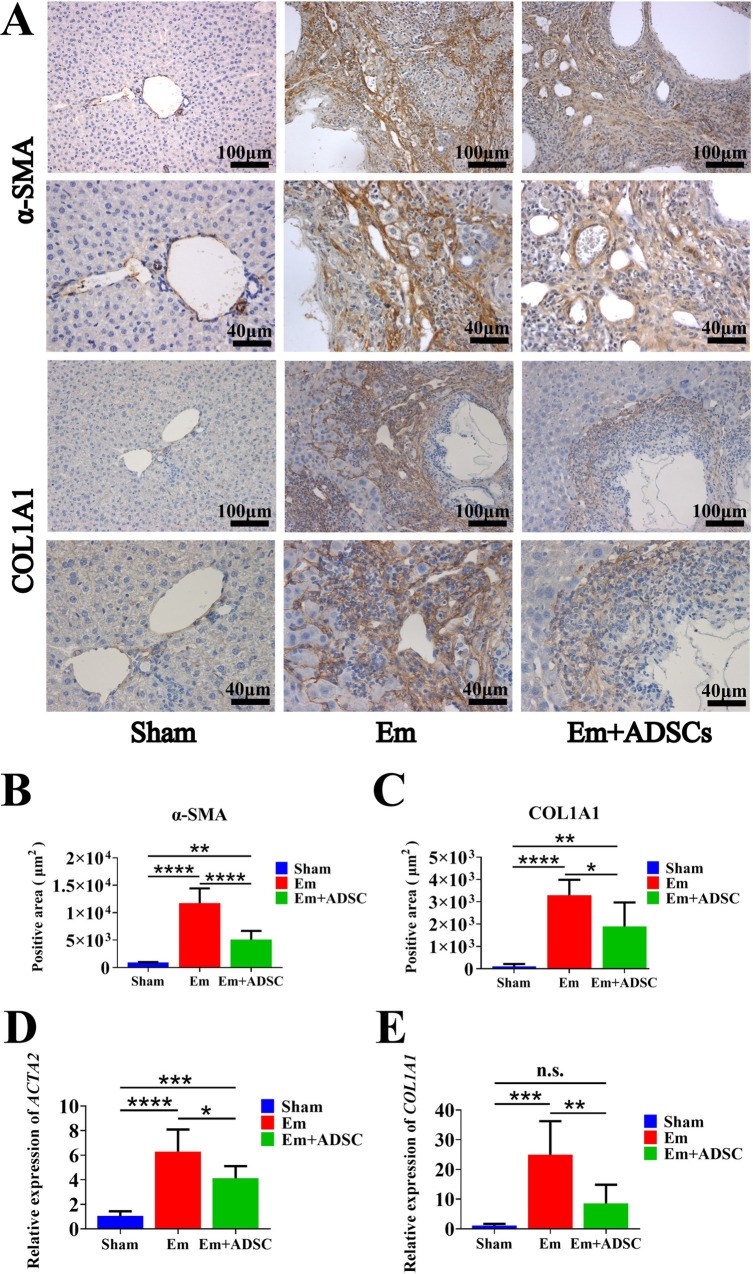
ADSCs transplantation inhibited HSCs activation and collagen deposition in *E*. *multilocularis* infected mice. A: Immunohistochemical staining of α-SMA and COL1A1. B: Quantification result of α-SMA expression. C: Quantification results of COL1A1 expression. D: Quantification results of *ACTA2* mRNA relative expression. E: Quantification results of *COL1A1* mRNA relative expression. Data are presented as mean ± SD (n = 6). n.s., no significance, *p < 0.05, ** *p* < 0.01, *** *p* < 0.001, **** *p* < 0.0001.

Thirty days after ADSCs transplantation, IHC and qRT-PCR were performed to assess the expression levels of α-smooth muscle actin (α-SMA) and collagen type I alpha 1 (COL1A1). IHC staining showed that the Em group had significantly higher levels of α-SMA and COL1A1 than the Sham group, while the Em + ADSCs group had significantly low α-SMA and COL1A1 levels than the Em group (**Fig 5A and 5B**). Compared to the Sham group, mRNA expression levels of *ACTA2* and *COL1A1* were significantly elevated in liver tissues of the Em group, while these gene expressions were effectively suppressed following ADSCs transplantation in the Em + ADSCs group (**Fig 5C**). In summary, ADSCs transplantation inhibited *E*. *multilocularis* infection induced HSCs activation and collagen secretion.

### ADSCs transplantation suppressed the TGF-β receptors expressions and up-regulated Smad7 level in *E*. *multilocularis* infected mice

**Fig 6:**
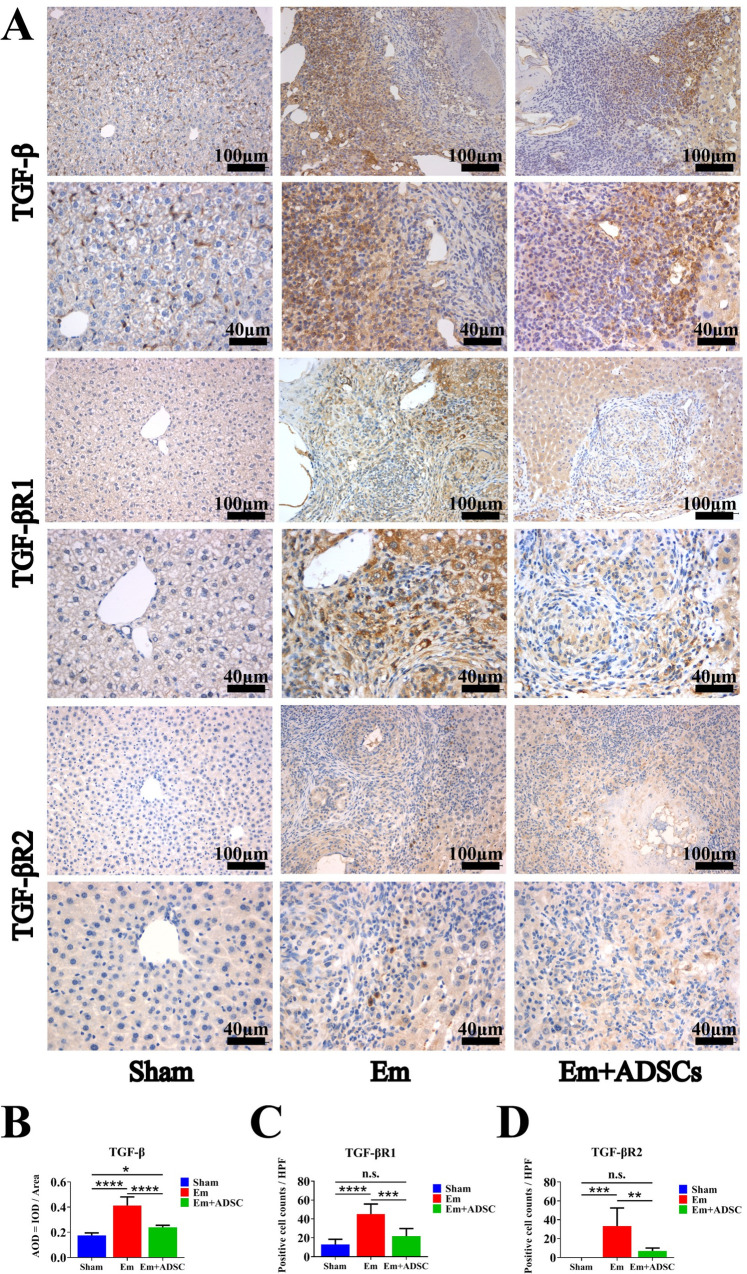
ADSCs transplantation inhibited TGF-β and TGF-β receptors expression in *E*. *multilocularis* infected mice. A: Representative immunohistochemistry images of TGF-β, TGF-βR1 and TGF-βR2 in each group. B: Quantification results of TGF-β expression. C: Quantification results of TGF-βR1 expression. D: Quantification result of TGF-βR2 expression. Data are presented as mean ± SD (n = 6). n.s., no significance, * *p* < 0.05, ** *p* < 0.01, *** *p* < 0.001, **** *p* < 0.0001.

**Fig 7:**
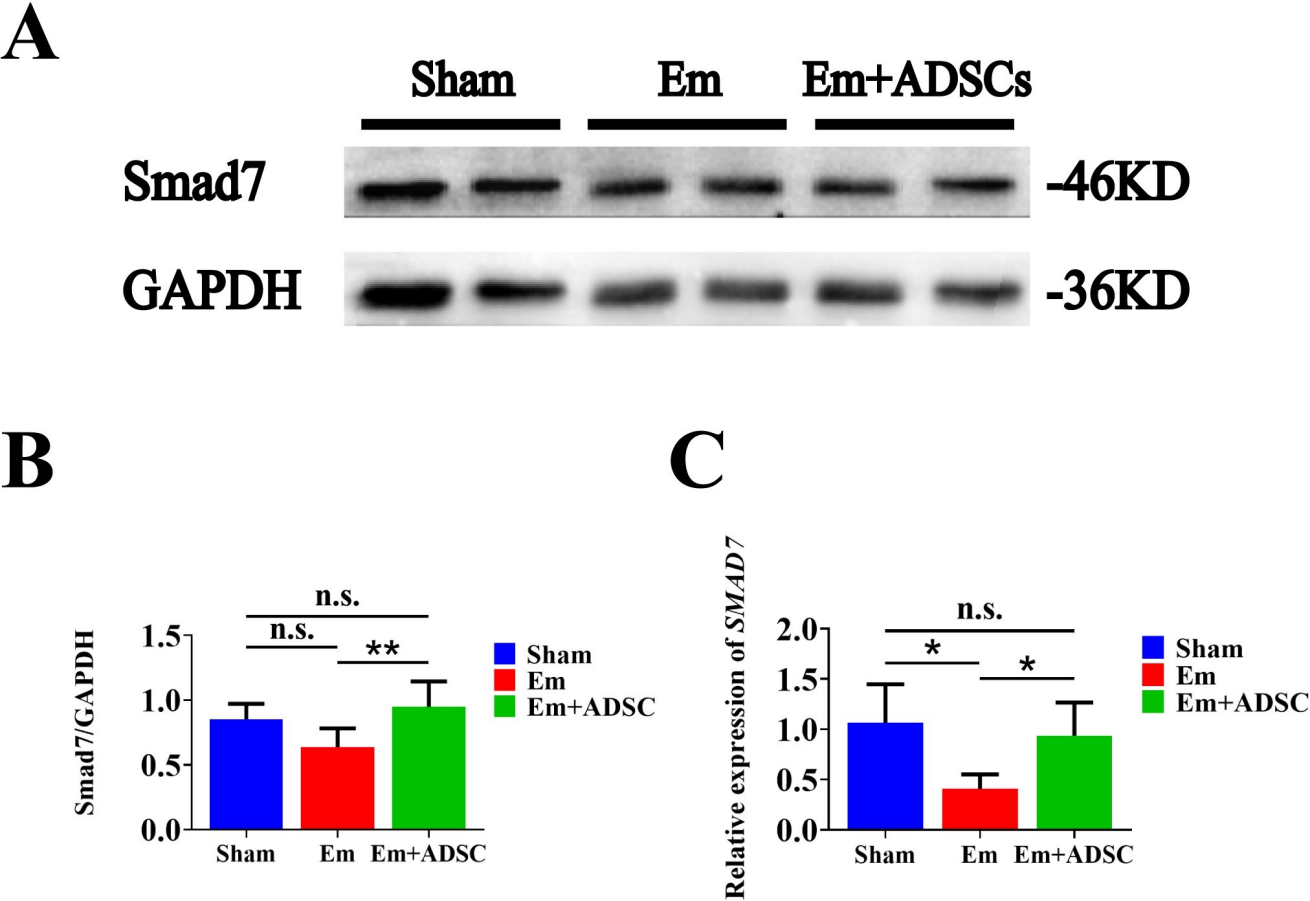
ADSCs transplantation up-regulated Smad7 expression in *E*. *multilocularis* infected mice. A: Western blotting results of Smad7. B: Quantification results of Smad7 protein expression. C: Quantification results of *Smad7* mRNA expression. n.s., no significance. Data are presented as mean ± SD (n = 6). *p < 0.05, **p < 0.01.

TGF-β expression was assessed by IHC thirty days after ADSCs transplantation. It was found that TGF-β expressions were significantly elevated in the Em group, relative to the Sham group. Moreover, relative to the Em group, TGF-β expression was significantly suppressed in the Em + ADSCs group following ADSCs transplantatio (**Fig 6A and 6B**). IHC was also performed to evaluate TGF-βR1 and TGF-βR2 expressions. Compared with the Sham group, TGF-βR1 and TGF-βR2 positive cells were significantly increased in Em and Em +ADSCs groups. Furthermore, compared to the Em group, the number of TGF-βR1 and TGF-βR2 positive cells in the Em +ADSCs group were decreased (**Fig 6A, 6C and 6D**). Smad7 expressions were assessed by Western blotting and qRT-PCR. Compared to the Sham group, expressions of Smad7 were suppressed in the Em group and restored to their normal levels in the Em +ADSCs group (**Fig 7**). These findings indicate that ADSCs transplantation alleviated liver fibrosis in *E*. *multilocularis* infected mice by modulating TGF-β/ Smad7 signaling.

## Discussion

In the present study, we used mice models of *E*. *multilocularis* infection to assess the therapeutic potential of ADSCs for AE. It was found that *E*. *multilocularis* infection activated hepatic stellate cells (HSCs) and increased collagen deposition around the lesion, both of which contributed to severe liver fibrosis, whereas ADSCs transplantation effectively relieved liver damage and fibrosis in *E*. *multilocularis* infected mice. Furthermore, ADSCs transplantation regulated the activation levels of the TGF-β receptors and Smad7 signaling pathway in the livers of *E*. *multilocularis* infected mice, suggesting that ADSCs rescue liver fibrosis by up-regulating Smad7 signaling.

Fibrosis is a histological hallmark of Alveolar echinococcosis (AE), which may result in impaired architectonics of the liver parenchyma or the death of the metacestode [30]. Studies have evaluated the effects of ADSCs on fibrosis in animal models or cells [31–33]. Shen et al. [34] showed that exosomal miR-19a from ADSCs inhibits TGF-β/Smad3 and p53 pathways by down-regulating HIPK2 levels, decreasing cell viability, and promoting ECM degradation, as well as by inhibiting the differentiation of corneal keratinocytes into myofibroblasts. Ma et al. [35] reported that ADSCs conditioned media inhibited the proliferation of fibroblasts derived from human hypertrophic scar in a dose-dependent manner via HGF-like protein, thereby playing critical roles in anti-fibrosis. However, the effects of ADSCs on AE-induced liver fibrosis have not been investigated. In this study, ADSCs were isolated from the mouse inguinal adipose tissue, which possesses the following characteristics: i. Adhesion ability; ii. Expression of MSCs lineage markers; and iii. Potential for differentiation. After CM-DiI labeling, the obtained ADSCs were able to stably proliferate and retain their original activities. One day after establishment of *E*. *multilocularis* infection models, ADSCs were intravenously administered. Consistent with previous studies, ADSCs transplantation resulted in a more regular arrangement of liver parenchymal structures, and decreased the fibrotic areas of liver tissue sections, implying that ADSCs transplantation can ameliorate liver fibrosis formation.

ADSCs exert anti-fibrotic effects through various mechanisms, including paracrine, immune regulation, or transdifferentiation into specific cell populations [13,19,36]. Hepatic stellate cells (HSCs), the main source of extracellular matrix proteins and main contributors to liver fibrosis are quiescent in normal livers. However, in damaged livers, quiescent HSCs transdifferentiate into proliferative myofibroblastic/activated HSCs [37]. Yu et al. [15] found that co-culturing ADSCs with HSCs for 72 h inhibited HSCs proliferation, activation, and suppressed collagen secretion of HSCs. In a previous study, protein and gene levels of α-SMA in AE patients were found to be significantly elevated in liver tissues adjacent to the lesion than in liver tissues distal to the lesion [38]. Consistent with previous studies, we found that as a marker of HSCs activation, α-SMA was significantly increased in the liver tissue around the lesions, suggesting that *E*. *multilocularis* infection activated HSCs. Expression levels of α-SMA were partially suppressed after ADSCs transplantation. Moreover, ADSCs transplantation partially alleviated the increase in collagen secretion induced by *E*. *multilocularis* infection.

Transforming growth factor-β (TGF-β) is a major profibrogenic cytokine that modulates HSCs activation and extracellular matrix homeostasis [39]. In addition to maintaining immune tolerance, TGF-β has been implicated in the development of fibrosis in AE [40]. It has been shown that ADSCs transplantation alleviates fibrosis via the TGF-β/Smad signaling pathway. Li et al. [41] showed that ADSCs-derived exosomes inhibited the proliferation and migration of hypertrophic scar-derived fibroblasts (HSFs). Furthermore, miR-192-5p contained in exosomes inhibits the expressions of Il-17RA in HSFs, as well as the phosphorylation of Smad2/3, thereby reducing fibrosis and promoting wound healing. Gao et al. [42] found that ADSCs-derived extracellular vesicles decreased PM2.5-induced apoptosis and necrosis in rat type 2 alveolar epithelial cells, and reduced lung injury. In addition, let-7d-5p, derived from extracellular vesicles, may inhibits TGF-βRI expression, thereby minimizing pulmonary fibrosis. Liao et al. [17] reported that ADSCs transplantation inhibited liver fibrosis in type 2 diabetic rats by down-regulating TGF-β1 expression and Smad3 phosphorylation, suggesting that ADSCs can also be used to treat liver fibrosis. However, it has not been determined if ADSCs exert their effects on AE-induced fibrosis by regulating the TGF-β signaling pathway. In this study, TGF-β1 levels were found to be elevated in liver tissues around the lesion in *E*. *multilocularis* infected mice. TGF-βR1 and TGF-βR2 positive cells were observed in periparasitic regions of *E*. *multilocularis* infected mice, but not in normal mice. Up-regulated TGF-β1 and down-regulated Smad7 in *E*. *multilocularis* infected mice were partially abolished by ADSCs transplantation, indicating that ADSCs regulate the TGF-β/Smad7 signaling pathway to ameliorate liver fibrosis. This study offers evidence for cellular and molecular interactions between the TGF-β signaling pathway and stem cells, highlighting the importance of stem cells in profibrogenic signaling. Elucidation of the involved mechanisms will form the basis for development of new treatment approaches for AE-induced fibrosis.

However, many questions remain unanswered. Firstly, single transplantation of ADSCs decreased the degree of fibrosis in the early-stage of *E*. *multilocularis* infection. However, it was not determined whether ADSCs can reverse fibrosis in the middle or end-stages of *E*. *multilocularis* infections. Secondly, ADSCs transplantation decreased the activation levels of the TGF-β and TGF-β receptors in the fibrotic liver. However, the precise cellular location of the affected TGF-β/Smad7 signaling pathway was not determined, and further researches should be performed to confirm target cells affected by ADSCs. In addition to HSCs, there are many cellular sources of TGF-β in the liver, including liver sinusoidal endothelial cells, macrophages, and hepatocytes [43,44]. Clarifying the cellular sources of TGF-β will inform on the mechanisms of ADSCs on liver fibrosis and establish the foundation for future clinical applications of ADSCs transplantation therapy. Thirdly, Kupffer cells (KCs), which are involved in the pathogenesis of chronic liver injury, are potential anti-fibrosis targets. KCs activate HSCs through a paracrine process that involves effective pro-fibrotic cytokines TGF-β [45]. In addition, bone marrow mesenchymal stem cell transplantation was shown to inhibit HSC activation by regulating the conversion of M1 macrophages to M2 macrophages, thereby reducing liver fibrosis [46]. Further research is needed to determine if ADSCs transplantation affects the TGF-β signaling pathway directly or indirectly, such as by regulating the functions of KCs.

## Conclusions

In conclusion, ADSCs transplantation alleviated liver fibrosis in *E*. *multilocularis* infected mice by up-regulating Smad7 and down-regulating the expressions of TGF-β receptors, the activation of HSCs, and collagen deposition. For the first time, we show that ADSCs transplantation decreased *E*. *multilocularis*-induced liver fibrosis, opening up a new aspect for ADSCs therapeutic applications. The mechanisms behind this finding should be studied to realize the therapeutic applications of ADSCs in AE.

## Supporting information

S1 Table Antibodies used in flow cytometry analysis. (DOC)

S2 Table Primer sequences used in this study. (DOC)

S1 Fig Representative images of metacestode tissues in the livers of mice from different groups. Metacestode tissues are encircled by the yellow line.
(TIF)
