# Supplementary material for: Transplantation of adipose-derived stem cells ameliorates Echinococcus multilocularis-induced liver fibrosis in mice
Source: PLoS Negl Trop Dis. 2022 Jan 31;16(1):e0010175. doi: 10.1371/journal.pntd.0010175 (PMC8830670; doi:10.1371/journal.pntd.0010175)
Supplement: S1 Table — (DOC) [file pntd.0010175.s001.doc]

**S1 Table.** Antibodies information used in flow cytometry analysis.

| **Target** | **Species** | **Conjugated fluorophore** | **Catalog number** | **Clone number** | **Supplier** |
| --- | --- | --- | --- | --- | --- |
| CD29 | Armenian Hamster Anti-mouse | FITC | 102205 | HMβ1-1 | Biolegend |
| CD90 | Rat Anti-Mouse | APC-Cy7 | 561641 | 53-2.1 | BD |
| CD105 | Rat Anti-Mouse | BB515 | 564744 | MJ7/18 | BD |
| CD44 | Rat Anti-Mouse | PE-Cy5 | 103009 | IM7 | Biolegend |
| CD31 | Rat Anti-Mouse | APC | 551262 | MEC 13.3 | BD |
| CD34 | Rat Anti-Mouse | FITC | 11-0341-82 | RAM34 | eBioscience |
| CD45 | Rat Anti-Mouse | APC | 559864 | 30-F11 | BD |
