# Supplementary material for: Transplantation of adipose-derived stem cells ameliorates Echinococcus multilocularis-induced liver fibrosis in mice
Source: PLoS Negl Trop Dis. 2022 Jan 31;16(1):e0010175. doi: 10.1371/journal.pntd.0010175 (PMC8830670; doi:10.1371/journal.pntd.0010175)
Supplement: S2 Table — (DOC) [file pntd.0010175.s002.doc]

**S2 Table. Sequence information for primers used in the study.**

| Gene | Species | Forward primer | Reverse primer |
| --- | --- | --- | --- |
| *COL1A1* | mouse | GCTCCTCTTAGGGGCCACT | ATTGGGGACCCTTAGGCCAT |
| *ACTA2* | mouse | GTCCCAGACATCAGGGAGTAA | TCGGATACTTCAGCGTCAGGA |
| *SMAD7* | mouse | CTGTGTTGCTGTGAATCTTACG | GAGACTCTAGTTCACAGAGTCG |
| *GAPDH* | mouse | AGGTCGGTGTGAACGGATTTG | TGTAGACCATGTAGTTGAGGTCA |
