# Supplementary figures and images for: Transplantation of adipose-derived stem cells ameliorates Echinococcus multilocularis-induced liver fibrosis in mice
Source: PLoS Negl Trop Dis. 2022 Jan 31;16(1):e0010175. doi: 10.1371/journal.pntd.0010175 (PMC8830670; doi:10.1371/journal.pntd.0010175)

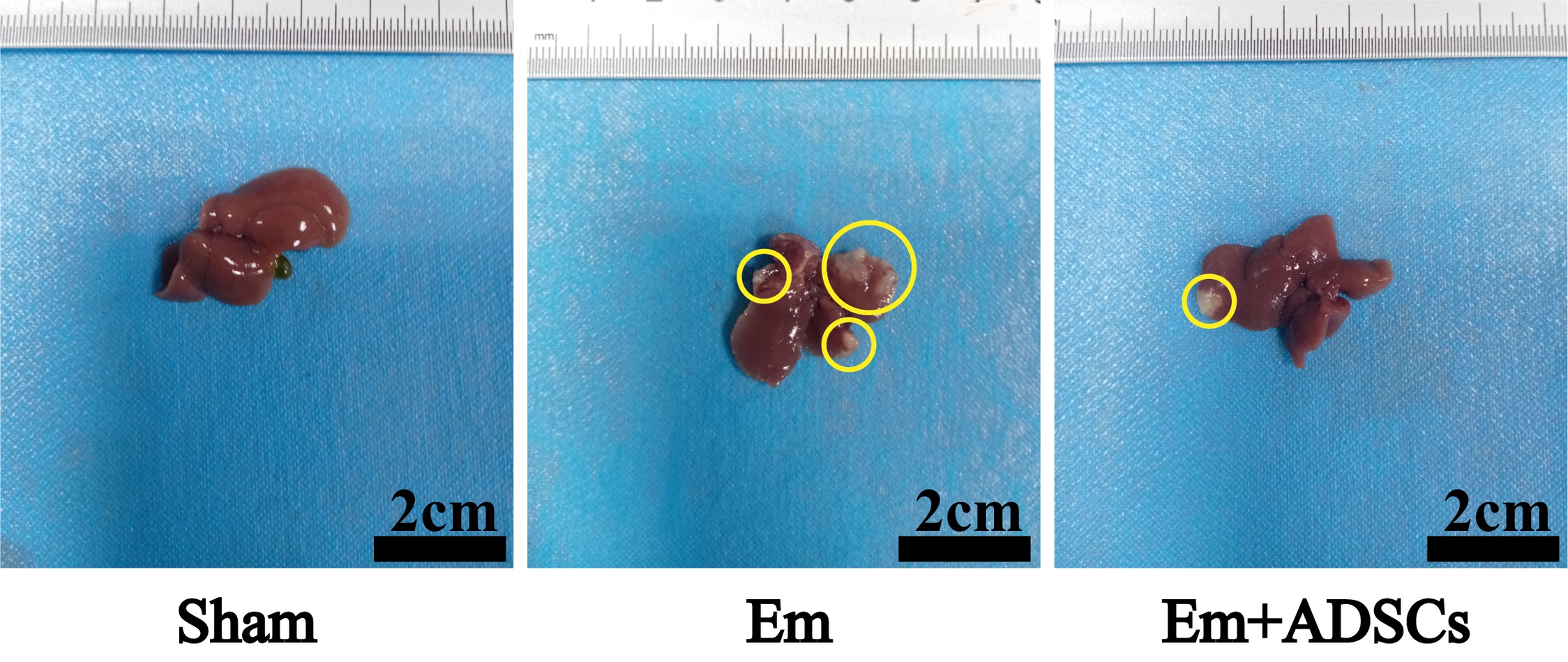

Supplement: S1 Fig — Metacestode tissues are encircled by the yellow line. (TIF) [file pntd.0010175.s003.tif]
